# High-resolution respirometry of fine-needle muscle biopsies in pre-manifest Huntington’s disease expansion mutation carriers shows normal mitochondrial respiratory function

**DOI:** 10.1371/journal.pone.0175248

**Published:** 2017-04-13

**Authors:** Eva Buck, Martina Zügel, Uwe Schumann, Tamara Merz, Anja M. Gumpp, Anke Witting, Jürgen M. Steinacker, G. Bernhard Landwehrmeyer, Patrick Weydt, Enrico Calzia, Katrin S. Lindenberg

**Affiliations:** 1 Department of Neurology, Ulm University, Ulm, Germany; 2 Division of Sports- and Rehabilitation Medicine, Ulm University Medical Center, Ulm, Germany; 3 Department of Neurodegenerative Diseases, Bonn University, Bonn, Germany; 4 Institute of Anesthesiological Pathophysiology and Process Development, Ulm University, Ulm, Germany; Rutgers University Newark, UNITED STATES

## Abstract

Alterations in mitochondrial respiration are an important hallmark of Huntington’s disease (HD), one of the most common monogenetic causes of neurodegeneration. The ubiquitous expression of the disease causing mutant huntingtin gene raises the prospect that mitochondrial respiratory deficits can be detected in skeletal muscle. While this tissue is readily accessible in humans, transgenic animal models offer the opportunity to cross-validate findings and allow for comparisons across organs, including the brain. The integrated respiratory chain function of the human vastus lateralis muscle was measured by high-resolution respirometry (HRR) in freshly taken fine-needle biopsies from seven pre-manifest HD expansion mutation carriers and nine controls. The respiratory parameters were unaffected. For comparison skeletal muscle isolated from HD knock-in mice (Hdh^Q111^) as well as a broader spectrum of tissues including cortex, liver and heart muscle were examined by HRR. Significant changes of mitochondrial respiration in the HdhQ knock-in mouse model were restricted to the liver and the cortex. Mitochondrial mass as quantified by mitochondrial DNA copy number and citrate synthase activity was stable in murine HD-model tissue compared to control. mRNA levels of key enzymes were determined to characterize mitochondrial metabolic pathways in HdhQ mice. We demonstrated the feasibility to perform high-resolution respirometry measurements from small human HD muscle biopsies. Furthermore, we conclude that alterations in respiratory parameters of pre-manifest human muscle biopsies are rather limited and mirrored by a similar absence of marked alterations in HdhQ skeletal muscle. In contrast, the Hdh^Q111^ murine cortex and liver did show respiratory alterations highlighting the tissue specific nature of mutant huntingtin effects on respiration.

## Introduction

Huntington’s disease (HD) is a neurodegenerative disorder caused by a CAG trinucleotide repeat expansion in the huntingtin gene [1]. So far, the biochemical downstream consequences of the presence of mutant huntingtin (mHTT) gene products in different cell types remain to be established. A widely accepted notion is that the expression of mHTT results in some changes of mitochondrial bioenergetics [2, 3] potentially not restricted to the neurological system [4]. In particular, functional changes in metabolically active organs (e.g. liver, pancreas) as well as skeletal muscle atrophy are observed in HD patients [5–7]. These alterations contribute to the characteristic weight loss in HD patients, which occurs despite high or increased food intake. [8, 9]. These metabolic symptoms are detectable in early stages of the disease and even in Huntington’s disease expansion mutation carriers (HDEMCs) [10].

Unfortunately, functional biomarkers to monitor the emergence and progression of metabolic alterations are not yet established. Markers of mitochondrial function are discussed as promising biomarker candidates. In different stages of the disease compromised mitochondrial function has been reported [11, 12] and the mitochondrial alterations probably emerge rather early, even in the pre-symptomatic stage [2, 13–15]. A multiplicity of techniques was used to identify numerous changes in mitochondrial morphology and function in HD [12, 16]. Most of these data were obtained from cellular and animal models for HD or from human post mortem tissue and therefore are limited to understand early mitochondrial changes caused by mHTT especially in peripheral tissue. Markers of mitochondrial function might pose an important opportunity to monitor disease progression in HD. As recently shown by Silva et al., for example, the enzymatic activity of the mitochondrial respiratory chain complexes is reduced in platelets from pre-symptomatic HD mutation carriers [17]. Similarly, a previous study by Saft et al. suggested that mitochondrial respiration in skeletal muscle is already compromised in HDEMCs, despite the absence of diagnostic motor signs [13]. However, the methods applied in the latter study do not allow to specifically identify alterations in the respiratory chain. It is unclear whether the observed differences between HDEMCs and healthy controls are based on an enzymatic defect of the mitochondrial respiratory chain. The differences could also result from defects in the capacity of ATP-synthesis or in the oxygen supply to the tissue [18]. Similarly, a reduction in the enzymatic activity of selective complexes of the respiratory chain as observed by Silva et al., does not necessarily imply a measurable limitation of mitochondrial respiration at the level of whole, undissociated mitochondria [17]. In fact, according to the metabolic control theory a specific threshold has to be exceeded for any single component to induce an overall limitation of the mitochondrial respiratory activity [19]. So far, the respiratory activity of whole, undissociated mitochondria from pre-manifest mutation carriers has not yet been studied. Identifying possible changes in mitochondrial function in pre-manifest HDEMCs is important to better understand a possible role of mitochondrial dysfunction in HD.

For several reasons, skeletal muscle is of interest for biomarker development investigating potential biochemical markers of HD, including early stages of HD. First, several molecular and physiological alterations have been reported in the skeletal muscle of pre-manifest HDEMCs [20, 21]. Second, skeletal muscle is accessible by biopsy in humans, thus allowing for biochemical studies of HD tissues. Third, being involved in overall metabolism, skeletal muscle is expected to provide specific insights into the development of metabolism-related HD symptoms, such as weight loss and an overall HD-associated increase in metabolism. Finally, in the R6/2 mouse model of HD parallel changes in muscle and brain have been shown at the level of gene expression (Luthi-Carter et al., 2002). Accordingly, skeletal muscle may serve as a mirror of mitochondrial dysfunction in the brain of HDEMCs.

In this study we analyze mitochondrial respiration in ex-vivo skeletal muscle tissue obtained by fine-needle muscle biopsies in a small group of HDEMCs and healthy controls. Human muscle biopsies were investigated using high-resolution respirometry with a well-established protocol [22, 23]. With this method the integrated function of the respiratory chain system in whole mitochondria can be determined in homogenized muscle fibers. Fine-needle muscle biopsies are minimally invasive, well tolerated and repeatable in patients. The technique described here may represent a promising approach to evaluate treatment effects in HD aiming to restore metabolic compromises. Finally we explored mitochondrial respiratory function in a disease stage comparable to pre-manifest HDEMCs in a transgenic HD mouse model. In addition to skeletal muscle tissue, samples of brain, liver and heart muscle, which are not easily accessible in humans, were studied in the HD Hdh^Q111^ mouse model. We aimed to evaluate to which extent mitochondrial respiration in skeletal muscle is comparable to other tissues with a special focus on the brain.

## Material and methods

### Human biopsies

The work with human subjects in this study was carried out with approval from the ethics committee of Ulm University (Nr. 78/14) in compliance with the guidelines of the federal government of Germany and the declaration of Helsinki. Muscle samples were taken from the middle of the belly of the right vastus lateralis muscle by a fine-needle biopsy technique using a 14G biopsy needle and a 14G puncture cannula (Pflugbeil, Zomeding, Germany) after disinfection and local anesthesia. Muscle tissue of about 4–5 mg was immediately transferred to the experiments within 45 min after biopsy. Muscle biopsies of nine control persons (four male, five female) aged 26–69 years with a mean of 33.3 years (SD ±9.7) and seven pre-symptomatic HDEMCs (stage 0, three female, four male) aged 23–74 years with a mean of 38.2 years (SD ±14.4) were analyzed by high resolution respirometry. Citrate synthase analysis was performed on the the basis of five controls and two HDEMCs.

### Animals

All experiments were performed on heterozygous Hdh^Q111/+^ knock-in mice and Hdh^Q20/+^ on a 129SvEv/CD1 background [24] (n = 6 for each group). Breeder pairs and oligonucleotides for the validation of genotypes were generously provided by Dr. Marcy MacDonald and Dr. Vanessa Wheeler (Massachusetts General Hospital, Boston, USA). A mouse colony was set up in the animal facility of Ulm University. Mice grew under approved conditions of the animal facility of Ulm University and the regional administrative council (Regierungspräsidium Tübingen). All animal experiments were conducted according to the protocol approved by the Institutional Animal Care and Use Committee of Ulm University (Tierforschungszentrum, Universität Ulm, and the Regierungspräsidium Tübingen) with the project license C/0.113. Handling of all animals was in accordance with the local regulations of the animal welfare committee. Mice were sacrificed by cervical dislocation by well trained personnel according to the EU guideline EURL 63/2010. Tissues of interest were harvested immediately. Tail biopsies were used for genotyping using the primer pairs indicated in Table 1.

**Table 1:** Primers for genotyping HdhQ knock-in mice.

Full name	forward	reverse
Hum: 31329_htt_h_F 33934_htt_h_R	ATGAAGGCCTTCCACTCCCTCAACTCCTTC	GGCGGCTGAGGAAGCTGAGGA
Mouse:31329_htt_ms_F 33934_htt_ms_R	GATGAAGGCTTTCGAGTCGCTCAAGTCGTTT	GGCGGCTGAGGGGGTTGAGGC

The human and the murine HTT were determined by PCR with the following program: 94°C for 1:30 min, 35 cycles of 94°C for 30 s, 65°C for 30 s and 72°C for 1:30 min, 72°C for 10:00 min. Male mice were used at an age of 450–484 days with an average of 471 days and sacrificed by cervical dislocation. Small biopsies of liver, soleus muscle, cortex and heart were immediately stored in ice-cold Custodiol^®^ (Dr. Franz Köhler Chemie GmbH, Bensheim, Germany) and further processed according to the protocols for high-resolution respirometry. All remaining parts of the tissues were snap-frozen in liquid nitrogen and stored at -80°C for further analysis.

### High-resolution respirometry

Mitochondrial respiration was measured in homogenized tissue of cortex, liver, heart and soleus muscle of the mice and in homogenized human muscle biopsies of the vastus lateralis muscle by means of high-resolution respirometry using the Oroboros^®^ Oxygraph-2K (Oroboros Instruments, Innsbruck, Austria). This device allows to simultaneously record the O_2_ concentration in two parallel chambers calibrated for 2 ml of respiration medium comprising sucrose, K-lactobionate, ethylene glycol tetra acetic acid, bovine serum albumin free from essentially fatty acids, MgCl_2_, taurine, KH_2_PO_4_, HEPES, adjusted to pH 7.1 with KOH and equilibrated with 21% O_2_ in N_2_ at 37°C. Mitochondrial respiration was quantified in terms of oxygen flux (*J*O_2_) based on the rate of change of the O_2_ concentration in the chambers normalized for wet tissue volume.

The homogenates of murine cortex, liver, heart and soleus muscle were generated from 20–30 mg of wet murine tissue and 4–5 mg human vastus lateralis muscle tissue suspended in 2 ml of ice-cold respiration medium using a potter S homogenizer (B.Braun, Melsungen, Germany). Aliquots of the homogenates were added to each oxygraph chamber in order to obtain a final amount of 1.25–2 mg tissue per chamber. Every sample was measured in duplicates and normalized to the amount of tissue per chamber.

**Fig 1:**
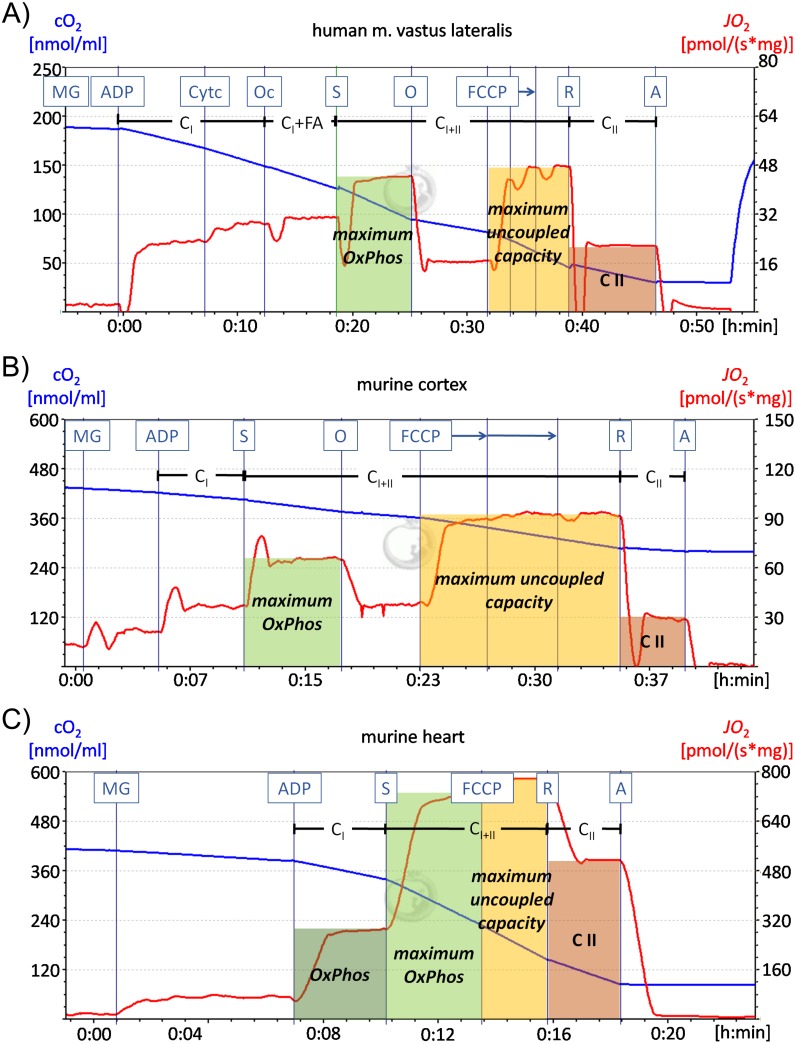
Representative high-resolution respirometry recordings of the human vastus lateralis muscle and the murine prefrontal cortex and heart. Representative measurements of high-resolution respirometry recordings are shown for the human vastus lateralis muscle (A), the murine prefrontal cortex (B) and the murine heart (C). The blue line represents the oxygen concentration in the chamber, while the red line indicates the oxygen flux. (A) For experiments with human vastus lateralis muscle samples 10 mM pyruvate were added before the recording started. After administration of 5 mM malate (M), 10 mM glutamate (G) and 5 mM ADP complex I activity (CI) was recorded and the leak respiration was calculated (respiration after MG/respiration after ADP). 10 μM cytochrome c was added to survey the integrity of the outer mitochondrial membrane. 1 mM octanoyl carnitine was administered to measure the fatty acid induced respiration. Further addition of 10 mM succinate, which is the complex II substrate, enables the determination of the maximum OxPhos capacity. The ATP synthase inhibitor oligomycin (O) (5 μM), which was not used for the murine heart (C) and soleus muscle was administered. After adding the uncoupling agent carbonyl cyanide p-(trifluoromethoxy)-phenylhydrazone (0.5, 0.25, 0.25 μM FCCP) the maximum uncoupled capacity was measured. Finally complex I was inhibited by rotenone (R) (0.5 μM) allowing the determination of complex II activity (CII). In the end complex III was inhibited by administration of antimycin A (5 μM). (B) The protocol for murine cortex and liver matched the human muscle protocol with the following exceptions: cytochrome c was added after pyruvate before the recording started and octanoyl carnitine was not used. (C) The protocol for murine heart and soleus muscle is equivalent to the one used for murine cortex (B) except for the oligomycin administration.

The titration sequence used for the human muscle samples was as follows: 10 mM pyruvate, 5 mM malate, 10 mM glutamate, 5 mM ADP, 10 μM cytochrome c, 1 mM octanoyl carnitine, 10 mM succinate 5 μM oligomycin, 0.5 μM carbonyl cyanide p-(trifluoromethoxy)-phenylhydrazone (FCCP), 0.5 μM rotenone and 5 μM antimycin A (Fig 1A). The *J*O_2_-values after ADP addition allow to selectively quantify the activity of complex I (*C*_*I*_). 10 μM cytochrome c was added to survey the integrity of the outer mitochondrial membrane. If homogenization steps damaged the mitochondrial membrane addition of cytochrome c induces an increase of the respiratory values. Octanoyl carnitine was added to the protocol adopted for the human muscle, where it yields a pronounced response when injected after the substrates for complex I. This step allows quantifying fatty acid supported changes in the respiratory capacity. The maximum oxidative capacity of the mitochondrial respiratory chain (*maximum OxPhos*) was determined after addition of the complex II substrate succinate. Subsequent injections of the ATP synthase inhibitor oligomycin and of the uncoupler FCCP allowed obtaining the maximum respiratory activity in the uncoupled state (*maximum uncoupled*). In both, human and murine tissues, a measure of the proton leak (*leak*) was obtained relating the respiratory activity after addition of malate and glutamate to the *J*O_2_ after injection of ADP. The ratio of the *maximum OxPhos capacity* to the *maximum uncoupled capacity* (*mO/mU*) with a value less than one indicates that oxidative phosphorylation is partially limited by the mitochondrial ATP-generation process. The O_2_ATP value is determined by subtracting the respiratory activity after oligomycine from the *maximum OxPhos capacity* to show the calculated oxygen consumption linked to the ATP production. Finally, the selective complex II activity (*C*_*II*_) was obtained at the end of the titration sequence by adding the complex I inhibitor rotenone in the maximum uncoupled state. In a final step complex III was inhibited by administration of antimycin A.

The titration scheme for murine cortex and liver mainly corresponds to the human protocol (Fig 1B). The protocol for measuring murine samples by high-resolution respirometry is well established in our lab and it is known that addition of octanoyl carnitine does not influence respiratory parameters in murine tissues. Therefore addition of octanoyl carnitine was omitted for the murine samples. In addition the murine protocol differs in the administration of cytochrome c, which was added directly after pyruvate. For the murine titration steps it is unusual to add cytochrome c before ADP and the response is more moderate in this case. But it is also important to add cytochrome c as early as possible, especially if a response is to be expected, because any titration step preceding the addition of cytochrome c is otherwise not comparable to later steps. In our case, the response to cytochrome c after addition of pyruvate was very small but still detectable. But cytochrome c addition early, while “normalising” the response between samples for better comparisons of early protocol steps, also precludes the possibility of comparing the outer membrane mitochondrial integrity between samples. Since in murine heart and soleus muscle adding FCCP after oligomycine does not re-establish full respiratory activity, the oligomycin step in the titration sequence in these two tissues was omitted (Fig 1C). Representative titration experiments are shown in Fig 1.

### Citrate synthase activity assay

The CS activity was determined by a spectrophotometric assay performed in a 96 well format in snap frozen aliquots of the tissue homogenates previously used for the high-resolution respirometry experiments [25]. In brief, the enzymatic activity of the CS was quantified at a temperature of 30°C by detecting the absorbance of thionitrobenzoic acid (TNB) generated by the transformation of acetyl-CoA, oxaloacetic acid and 5,5’-Dithiobis(2-nitrobenzoic acid) (DTNB) to citrate and TNB-CoA at a wave length of 412 nm. The reaction mix consisted of 0.25% Triton X-100, 0.31 mM acetyl-CoA, 0.1 mM DTNB dissolved in 1 M Tris-HCl-Buffer of pH 8.1, 10 μl sample for murine liver, heart and brain tissue homogenate, whereas 15 μl lysate were used of the murine soleus muscle and 25 μl of the human vastus lateralis muscle homogenates. 790 μl H_2_O and 0.5 mM oxaloacetate in 0.1 M triethanolamine-HCl-buffer of pH 8.0 were added as well. A porcine heart CS standard was used in each experimental run as reference. Each sample was measured in triplicate using 240 μl per well of the total mix containing 1000 μl using a Spectra Max190 spectrophotometer (Molecular Devices, Sunnyvale, CA). The absorbance was recorded in 20 s intervals for 15 min. The CS activities are reported in (μmol*mg)/min.

### Quantitative real-time-PCR

Total RNA was isolated by the RNeasy Plus Universal Kit (Qiagen, Hamburg, Germany) in accordance to the manufacturer’s instructions. Briefly, snap frozen tissue (cortex, liver, heart and gastrocnemius muscle) was thawed and homogenized in Quiazol with a TissueLyser (Qiagen, Germany) at 50 Hz for 2–5 min. An aqueous RNA including layer was separated by centrifugation in the presence of phenol and chloroform. Genomic DNA was removed by addition of the gDNA eliminator solution. The RNA concentration was determined by Nanodrop^®^ (Thermo Scientific, Waltham, MA) and 1 μg of total RNA was used for cDNA synthesis using iScript reverse transcriptase (Bio-Rad Laboratories, Munich, Germany) containing oligo-dT primers and random primers.

Ten times diluted cDNA was applied for PCR analysis on a Bio-Rad System using iQ^™^ SYBR^®^ Green Supermix (Bio-Rad Laboratories). In parallel, a standard curve was measured for each gene in a five-fold dilution series to assess the specific primer efficiencies. All reactions were performed in duplicates in a 96-well format on a CFX384 Touch Real-Time PCR Detection System (Bio-Rad Laboratories) with the following program: 95°C for 3 min, 40 cycles at 95°C for 15 s and a primer specific annealing temperature for 15 s, finally a 60°C to 95°C melting curve at increments with steps of 0.5°C every 5 s was determined.

The targets examined and the respective primers are listed in Table 2. CFX Manager software (Bio-Rad Laboratories) was used for analysis. Briefly, relative quantification was performed by calculating the ratio between the cycle numbers of the threshold within the logarithmic phase of the gene of interest relative to that of the reference genes. Methionyl aminopeptidase 1 (*Metap1*, polymerase (RNA) II (DNA directed) polypeptide A (*Polr2a*) and hypoxanthine guanine phosphoribosyl transferase (*Hprt*) were chosen as reference genes for heart muscle, brain and liver [26, 27]. For analysis of gastrocnemius muscle data *Metap1* and *Polr2a* were chosen as reference genes with a heterogeneous stability.

**Table 2:** Mouse primers for qPCR.

Full name	Symbol	Forward	Reverse	NCBI NM_ number	Efficiency [%]	[°C]
peroxisome proliferative activated receptor gamma coactivator 1 alpha	*Ppargc1a*	AGAGTGTGCTGCTCTGGTTG	TTCCGATTGGTCGCTACACC	008904.2	101.8	60
nuclear respiratory factor 1	*Nrf1*	GCTGCAGGTCCTGTGGGAAT	ACTCAAACACATGAGGCCGT	001164226.1	110.4	60
catalase	*Cat*	CCTTCAAGTTGGTTAATGCAGA	CAAGTTTTTGATGCCCTGGT	009804.2	87.0	60
superoxide dismutase 2	*Sod2*	GACCCATTGCAAGGAACAA	GTAGTAAGCGTGCTCCCACAC	013671.3	96.0	60
transcription factor A	*Tfam*	CAAAGGATGATTCGGCTCAG	AAGCTGAATATATGCCTGCTTTTC	009360.4	97.6	60
uncoupling protein 2	*Ucp2*	AGCCTGAGACCTCAAAGCAG	CCTTCAATCGGCAAGACG	011671.4	96.5	60
hypoxanthine guanine phosphoribosyl transferase	*Hprt*	GGAGCGGTAGCACCTCCT	CTGGTTCATCATCGCTAATCAC	013556.2	92.8	60
methionyl aminopeptidase 1	*Metap1*	TGCGACTCGTGTGTAGGC	CTTCAGTAGTTACACCCGCTTTAAT	175224.4	101.6	60
polymerase (RNA) II (DNA directed) polypeptide A	*Polr2a*	GCTGGGAGACATAGCACCA	TTACTCCCCTGCATGGTCTC	001291068.1	94.8	60

### Mitochondrial DNA copy number

The mtDNA copy number was determined based on a previous publication [27] using primers which were designed by primer-BLAST for a nuclear single copy gene and a stable region of the mitochondrial genome. The primer search was restricted to primers without exon-exon junction, an annealing temperature optimum of 60°C and to the species mus musculus (Table 3). Genomic and mitochondrial DNA was isolated using the Puregene Core Kit A (Qiagen, Hilden, Germany) according to manufacturer’s instructions. DNA was isolated of frozen tissue using 10 mg for brain, liver and heart, whereas about 5 mg of soleus muscle was used. After isolation, the DNA concentration and purity was determined using a Nanodrop^®^ (Thermo Scientific, Waltham, MA). 8 ng DNA was applied for the quantitative real-time PCR reaction. A DNA standard dilution series of two-fold dilutions starting at a concentration of 52 ng per well was prepared based on a mixture of all samples (1 μl each). The frequency of the nuclear single copy gene beta-2 microglobulin (B2M) was determined to normalize the frequency of the relatively stable mitochondrial displacement loop (D-loop). The primers used are listed in Table 3. All samples were measured in technical triplicates in a 96-well format on a CFX384 Touch Real-Time PCR Detection System (Bio-Rad Laboratories) using iQ^™^SYBR Green^®^ Supermix (Bio-Rad Laboratories) and the following program: 10 min at 95°C, 40 cycles of 10 s at 95°C, 15 s at 61°C, 20 s at 72°C, finally a 60–95°C melting curve was determined by increments of 1°C every 0.05 s [27]. Data is shown relative to the values obtained for Hdh^Q20^ mice in the cortex.

**Table 3:** Mouse primers used to amplify genomic and mitochondrial DNA.

Full name	Official symbol	forward	reverse	Temp. [°C]
displacement loop	Dloop	AGGCATGAAAGGACAGCACA	GGTGATTGGGTTTTGCGGAC	60
beta-2 microglobulin	B2M	GCTCACACTGAATTCACCCC	CGGCCATACTGGCATGCTTA	60

### Data analysis and statistics

All data are presented as box plots. The box represents the 25^th^ to 75^th^ percentile. The median is indicated with a line in the middle of the box. The whiskers show the smallest and largest values. Differences between controls and HDEMCs or between Hdh^Q20^ and Hdh^Q111^ were analyzed by the two-tailed student's t-test after confirmation of normal distribution using the Kolmogorov-Smirnov test. For every two-tailed student’s t-test an F-test was performed to test for eventual unequal variances. In case of significant differences in variance and for data which was not normally distributed the nonparametric Mann-Whitney Rank-test was used as indicated in the respective figure legend. Differences were considered statistically significant when p<0.05. Significance is indicated by ns = p>0.05, * = p≤0.05, ** = p≤0.01, *** = p≤0.001.

## Results

### High-resolution respirometry in the human muscle vastus lateralis muscle

In a human study cohort (Table 4) including HDEMCs and controls, the mitochondrial function was determined by high-resolution respirometry. To quantify the integrated mitochondrial respiratory capacity in human muscle biopsies and in different tissues of the Hdh^Q111^ HD mouse model compared to controls, we performed high-resolution respirometry in the human vastus lateralis muscle and the murine prefrontal cortex, liver, heart and skeletal muscle (soleus muscle). Representative recordings of mitochondrial respiration obtained by high-resolution respirometry of the freshly isolated tissues are delineated for human vastus lateralis muscle (Fig 1A), the murine prefrontal cortex (Fig 1B) and the murine heart (Fig 1C). The oxygen concentration (cO_2_) in the chamber was recorded (Fig 1, blue lines) to calculate the oxygen flux (JO_2_, Fig 1, red lines), which allows to conclude the oxygen consumption of the respiratory chain complexes. Application of different substrates and inhibitors for the respiratory chain complexes (Fig 1, boxes at the top) enabled to record data, illustrating the activity of separate components as well as overlapping functions of the respiratory chain complexes, drawing an integrated picture of the mitochondrial function.

**Table 4:** Clinical data of the human study cohort.

Gender	Mean age	Clinical information	HD stage	CAG-1	CAG-2	HRR	CS activity	Disease burden
♀	33.3±9.7	control				all	2x NA	
♂		control				all	all	
♀	38.2±14.4	HDEMC	PM	19.8±4.1	44.8±3.3	all	NA	391
♀		HDEMC	PM				NA	274.5
♀		HDEMC	PM				X	350
♂		HDEMC	PM				X	294.5
♂		HDEMC	PM				NA	264.5
♂		HDEMC	PM				X	315
♂		HDEMC	PM				X	259

The clinical data of the study cohort is shown according to gender, age, clinical information, Huntington’s Disease (HD) stage, CAG repeats, disease burden (= (CAGn-35.5) X age [28]), application of the sample for high-resolution respirometry (HRR) and citrate synthase (CS) activity, which was not possible to be determined for all samples, due to the small size of the biopsy, indicated by not available (NA).

**Fig 2:**
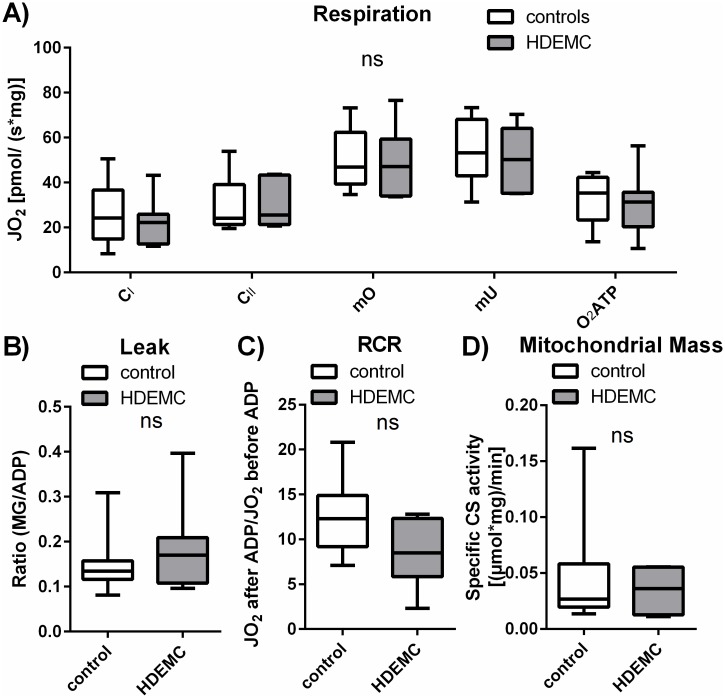
High-resolution respirometry of human muscle biopsies show no difference between mutation carriers and healthy controls. High-resolution respirometry analysis of human vastus lateralis muscle muscle biopsies. The white boxes represent human controls (n = 9) versus HD expansion mutation carriers (HDEMC) (gray boxes) (n = 7). (A) Respiratory function of complex I activity (CI), complex II activity (CII), the maximum OxPhos capacity (mO), the maximum uncoupled capacity (mU) and the O2ATP (calculated oxygen consumption linked to ATP production) are shown. The leak respiration is illustrated in (B). The respiratory control ratio (RCR) is indicated in (C). The mitochondrial mass, determined using a citrate synthase (CS) activity assay, is shown in (D) (n = 7 controls, n = 4 for HDEMCs). Mann-Whitney test comparing controls vs. HDEMCs, ns = P>0.05, * = P≤0.05.

In the human vastus lateralis muscle biopsies of HDEMCs and controls the respiratory parameters of *maximum OxPhos capacity* and *maximum uncoupled capacity* were determined after respective addition of succinate and carbonyl cyanide p-(trifluoromethoxy)-phenylhydrazone (FCCP). No significant changes between HDEMCs and controls were detected in these parameters (Fig 2A). In addition complex I and complex II activity were examined in the limited amount of tissue sample, by accepting the restriction that complex II activity could be determined in an uncoupled state only, as it was described before [22, 29]. Neither complex I activity nor complex II activity showed significant changes between the HDEMCs and the control group. The calculated value for the oxygen consumption, which is linked to the ATP production (Fig 2A, O_2_ATP) was unchanged between HDEMCs and controls. To analyze the leak respiration the quotient of the respiration measured after malate and glutamate by the respiration measured after ADP was determined. A trend towards slightly increased leak respiration was detected for HDEMCs (p = 0.59) (Fig 2B). The respiratory control ratio (RCR) represents the coupling state of the mitochondria, starting from 1 (fully dys-coupled) to infinity (fully coupled). The RCR was unchanged in the vastus lateralis muscle of HDEMCs compared to controls (p = 0.091), but a tendency to decreased values in the HDEMC samples was shown (Fig 2C). This could point out a higher degree of uncoupling in the HD mutation carriers, but further experiments will be necessary to confirm this trend. In addition to the mitochondrial activity, mitochondrial content was characterized by a CS activity assay. The CS activity and therefore the mitochondrial content did not differ significantly between the HDEMCs and the controls (Fig 2D). In addition to the absolute values of the respiratory capacities, respiration was also normalized to the CS activity, the *mU* (calculating the flux control ratio (FCR)) and the cytochrome c oxidase (COX) activity (S1 Fig). No significant changes between controls and HDEMCs were detected.

In summary the mitochondrial respiratory data of the human vastus lateralis muscle show no differences between the HDEMCs and the controls despite a tendency of decreased levels of the RCR in the vastus lateralis muscle of HDEMCs, indicating a more uncoupled status of mitochondria.

### High-resolution respirometry of murine cortex, liver, muscle soleus and the heart

**Fig 3:**
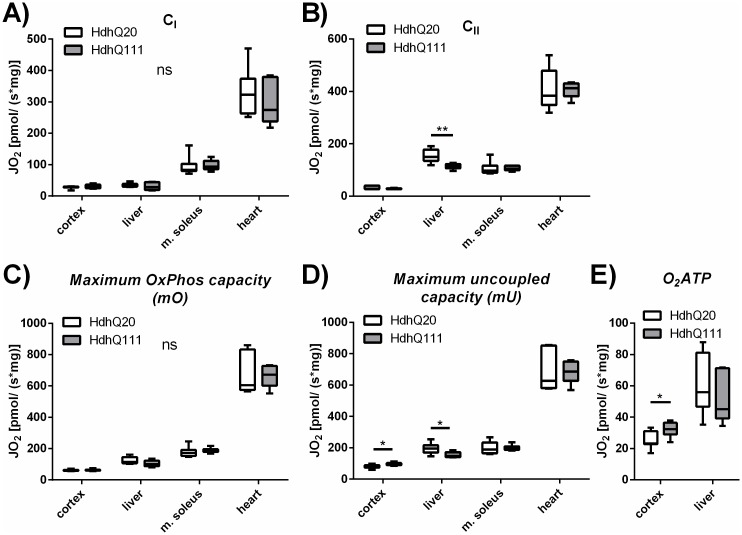
Respiration of cortex, liver, soleus muscle and heart of Hdh^Q20^ and Hdh^Q111^ mice. High-resolution respirometry in the murine tissues prefrontal cortex, liver, soleus muscle and heart. The white boxes represent Hdh^Q20^ control mice versus the gray boxes, which represent the HD mouse model Hdh^Q111^. (A) Complex I activity (C_I_) is shown in a coupled state determined after addition of ADP. (B) Complex II activity (C_II_) was measured in an uncoupled state after addition of rotenone. (C) The *maximum OxPhos* capacity was measured and (D) the *maximum uncoupled capacity* was determined after application of FCCP in all four tissues. (E) For murine cortex and liver the oxygen consumption linked to ATP production (O_2_ATP) was calculated. n = 6, student’s t-test comparing genotypes, ns = p>0.05, * = p≤0.05, ** = p≤0.01. 95% Confidence interval limits: (B) liver: 14.72–65.71; (D) cortex: 1.20–28.16; liver: 4.99–73.43.; (E) cortex: 0.96–13.67. The Mann-Whitney Rank-Test was used for the following samples: (A): cortex; (B): cortex, m. soleus, heart; (D): m. soleus.

The *maximum OxPhos* and *maximum uncoupled* capacity as well as complex I activity (*C*_*I*_) and complex II activity (*C*_*II*_*)* markedly differed in the four murine tissues, being highest in the heart and lowest in the cortex (Fig 3A–3D). The liver had the highest ratio of *C*_*II*_ to *C*_*I*_, suggesting a higher complex II activity. No significant changes in *C*_*I*_ *or C*_*II*_ between Hdh^Q111^ and Hdh^Q20^ mice could be determined in any tissue, despite the liver, where *C*_*II*_ was significantly decreased in Hdh^Q111^ mice compared to respective controls (Fig 3B). This was also reflected in a decreased *maximum uncoupled* capacity in the liver of the Hdh^Q111^ mice (Fig 3D). The *maximum OxPhos* was unchanged in all four tissues between the HD mouse model and control mice (Fig 3C). In contrast to the liver, a significantly increased *maximum uncoupled* capacity in cortex of the Hdh^Q111^ mice was observed (Fig 3D). In the cortex of Hdh^Q111^ mice the calculated O_2_ATP value was significantly increased compared to Hdh^Q20^ control mice, showing that the oxygen consumption linked to ATP production was higher in the HD mouse model (Fig 3E, p≤0.05). Further analysis of the data was performed and all respiratory values were normalized to the CS activity for normalization to the mitochondrial content (S2A–S2E Fig). No significant differences were detected. The respiratory data was also normalized to the *mU* respiration, where significantly decreased levels of the respiratory ratios were detected in the cortex of Hdh^Q111^ mice for *C*_*II*_ and the *mO* (S2F–S2I Fig).

Considering all respiratory data collected in different tissues of the Hdh^Q111^ mice, significant differences are based on changes in the liver and the cortex of the HD model compared to control mice.

### Unchanged mtDNA copy number in Hdh^Q20^ versus Hdh^Q111^ mice

**Fig 4:**
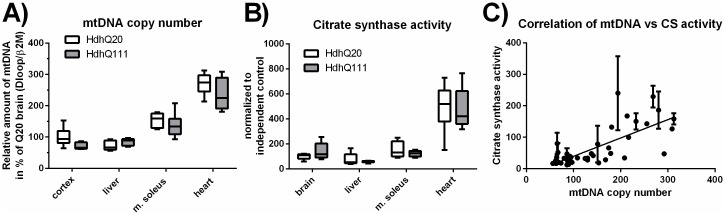
Mitochondrial mass in HdhQ mice in brain, soleus muscle, liver and heart. (A) The mtDNA copy number was determined by qPCR for the murine cortex, liver, soleus and heart muscle, comparing Hdh^Q20^ control mice (white boxes) to Hdh^Q111^ HD mice (gray boxes). n = 6–7. (B) The citrate synthase (CS) activity was measured by a spectrophotometric assay. n = 5, student’s t-test comparing genotypes of one tissue, ns = p>0.05. (C) Correlation of mtDNA copy number to the citrate synthase activity, which was determined in triplicates (±SD). Pearson value p≤0.001, R^2^ = 0.5371. The Mann-Whitney Rank-Test was used for the following samples: (B): cortex, liver.

To investigate, whether the different respiratory activities in the tissues were related to differences in mitochondrial mass, the mtDNA copy number was quantified (Fig 4A). The mtDNA copy number was highest in the heart showing an approximately 1.7 times higher level compared to soleus muscle, and even higher when compared to brain (2.9 times) and liver (3.2 times). However, there were no significant differences between control animals (Hdh^Q20^) and the disease mouse model (Hdh^Q111^) (Fig 4A). As mtDNA is only partially a valuable tool to quantify mitochondrial mass we determined a second marker for mitochondrial mass, the CS activity spectrophotometrically (Fig 4B). Similar to the mtDNA copy number, we found highest CS activity levels in the heart followed by the soleus muscle and the prefrontal cortex, while liver showed the lowest CS activity levels. No significant changes were detected between the Hdh^Q20^ and Hdh^Q111^ mice. Finally, the two markers of mitochondrial mass were found to correlate significantly (p<0.0001, Fig 4C), confirming the same tendency.

### mRNA levels of Sod2 and Ucp2 are differentially expressed in the brain of Hdh^Q111^ mice

To further characterize mitochondrial function, specific target genes important for mitochondrial biogenesis and energy metabolism were examined in the murine tissues on mRNA level. mRNA was isolated of snap frozen tissue samples of the same mice, which have been used for the high-resolution respirometry experiments as well as for determination of the mtDNA copy number and CS activity assays. For cortex, liver and heart the amount of tissue was sufficient to perform all experiments. Due to limitations in the amount of soleus muscle, the gastrocnemius muscle was chosen to be examined on mRNA level instead, allowing the examination of tissue from the same animals in all experiments. For all tissues mentioned, we determined the levels of the two ROS detoxifying enzymes catalase (*Cat*) and superoxide dismutase 2 (*Sod2*), as well as the levels of the nuclear respiratory factor 1 (*Nrf1*), peroxisome proliferator activated receptor gamma coactivator 1 alpha (*Ppargc1a*), transcription factor A (*Tfam*) and uncoupling protein 2 (*Ucp2*), which are involved in the energy metabolism of mitochondria. To analyze the data the student’s t-test was performed to compare the mRNA levels between the control (Hdh^Q20^) mice and the mice representing the disease model (Hdh^Q111^).

**Fig 5:**
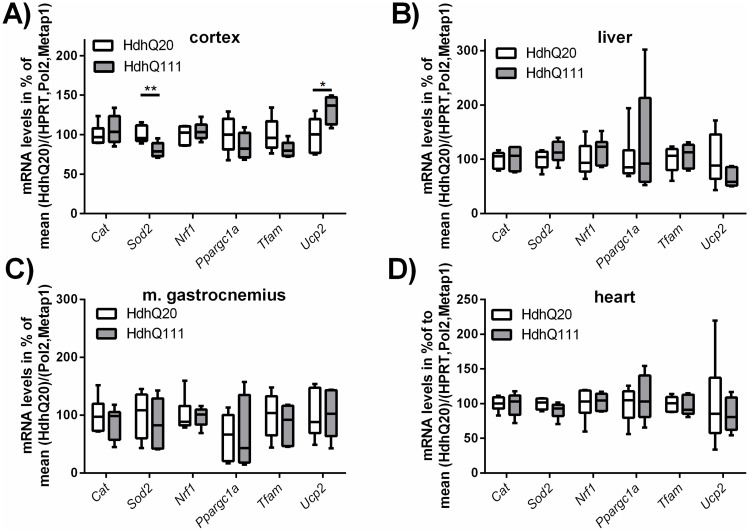
mRNA levels of mitochondrial energy metabolism related target genes and genes involved in ROS detoxification. mRNA analysis of mitochondrial targets in the murine tissues (A) cortex, (B) liver, (C) gastrocnemius muscle and (D) heart. mRNA levels of target genes are normalized to the reference genes (hypoxanthine guanine phosphoribosyl transferase (*Hprt*), polymerase (RNA) II (DNA directed) polypeptide A (*Polr2a*), methionyl aminopeptidase 1 (*Metap1*) for the cortex, liver, heart and *Polr2a*, *Metap1* for the gastrocnemius muscle) and the results are shown relative to the mean of Hdh^Q20^ values in box plots. n = 5–6, student’s t-test comparing genotypes of one tissue, ns = p>0.05, * = p≤0.05, ** = p≤0.01. 95% confidence interval limits: (A) Sod2: 6.24–32.35; Ucp2: 3.21–60.09. The Mann-Whitney Rank-Test was used for the following samples: (B) *Ppargc1a*, *Ucp2*; (D): *Ppargc1a*.

We found specific changes in these target genes only in the cortex (Fig 5). Here the *Sod2* levels were significantly reduced in Hdh^Q111^ mice, whereas *Ucp2* was upregulated (Fig 5A). All other examined tissues like liver (Fig 5B), gastrocnemius muscle (Fig 5C) and heart (Fig 5D) showed no significant changes in the mRNA levels of the analyzed targets.

## Discussion

In the present study we demonstrated the feasibility of human fine-needle muscle biopsies in HDEMCs for HRR. No differences in the mitochondrial respiration were observed in the human muscle biopsies and skeletal muscle of the HdhQ mouse model. In contrast mitochondrial respiration in liver and brain showed tissue specific changes in the knock-in mouse model HdhQ.

Several studies describe early disturbances in energy metabolism and mitochondrial function, but only few explored mitochondrial activity in pre-manifest HD [12, 16]. An impairment in mitochondrial respiration in HDEMCs is indicated by data on the enzymatic activity of the respiratory chain complexes in human platelets [17]. In another study by Saft et al, in-vivo experiments of the phosphocreatine recovery in the skeletal muscle using the ^31^P-NMR-technique were performed. In the latter study no differences were detected at rest, only after hypoxic exercise a possible mitochondrial dysfunction in the HDEMCs was suggested [13]. In our study the pre-symptomatic HDEMCs did not undergo any specific exercise before testing. Unchanged mitochondrial respiratory complex activity in the fine-needle muscle biopsies is in good agreement with the findings of Saft et al. This result is not unexpected given the absence of an overt clinical phenotype in this group. The differences between the in-vivo technique adopted by Saft et al. and our current ex-vivo approach based on the high-resolution respirometry are important for interpretation of the results. The ^31^P-NMR-technique partially depends on the oxygen availability due to tissue perfusion, which could also be disturbed in the HDEMCs, especially during or after exercise. Furthermore, the measurement of phosphocreatine recovery depends on the capacity of synthesizing ATP [18]. In contrast, our ex vivo data more specifically reflect the function of the mitochondrial respiratory chain and of the biochemical steps involved in ATP-production in whole mitochondria. In our study, mitochondrial activity did not show any difference between HDEMCs and healthy controls, neither in the coupled nor in the uncoupled state. Therefore, we can conclude that in HDEMCs the respiratory chain function and ATP production of intact mitochondria are not limiting factors yet for the aerobic metabolism of skeletal muscle at rest. Noteworthy, this conclusion does not exclude the possibility, that the enzymatic activity of some individual complexes of the mitochondrial respiratory chain may be already altered, as observed in human platelets by Silva and colleagues [17]. However, our results clearly preclude that any possible alterations of individual complexes of the mitochondrial respiratory chain are of an extent resulting in a reduction beyond the threshold required for a measurable limitation of the mitochondrial respiratory capacity. In summary, our data do not confirm the notion that there is a clear reduction of mitochondrial respiration in pre-manifest stages of HD. No significant changes in respiratory values were found after normalizing the data to CS activity, *mU* respiration or COX activity. For analysis of the CS activity, which represents the mitochondrial content, the number of the samples was restricted to n = 7 controls and n = 4 HDEMCs and therefore the respiratory analysis normalized to the CS activity is also restricted to these numbers. The reduced number of samples is due to the limited amount of the human fine-needle muscle biopsies, which also excluded the possibility for further experiments including a morphological analysis.

A limitation of our study is the small sample size with respect to the number of participants. In addition, the participants in this study were not matched for gender and the groups show a broad age range. Nevertheless, the variation of mitochondrial activity was rather low suggesting low physiological variability of the parameters measured. Maybe this also indicates the robustness of mitochondrial respiration and the ability of the mitochondria to compensate for respiratory deficits. The present study design was not aimed for the detection of age- or sex-specific differences between controls and HDEMCs. The main focus was to investigate the feasibility of the fine-needle muscle biopsies with subsequent analysis of the respiratory capacity in a group of different age and gender for controls and HDEMCs. The intention was to search for obvious differences over all ages and both genders to discover robust differences underlying all pre-manifest HDEMCs compared to controls. Gender-specific genetic modifiers of HD have been described earlier [30–32] and additionally it is known that the mitochondrial mass in muscle tissue declines with age due to sarcopenia [33–35]. On the bases of these reports respiratory changes would be expected with regard to differences in age and gender and should be examined in further experiments.

Skeletal muscle is increasingly affected during progression of HD [36] and in contrast to the brain relatively accessible and therefore of interest for biomarker development. At least in the R6/2 mouse model of HD, the HD-related dysregulation of gene expression found in the skeletal muscle has been suggested to reflect the same aspects of the disease pathology that was found simultaneously in the brain [37]. Therefore, studies of skeletal muscle in HD hold promises in the search for disease progression markers [4]. In the present study, we demonstrated the feasibility of fine-needle muscle biopsies in HDEMCs for high-resolution respirometry employing a procedure that was well tolerated and without withdrawals because of adverse effects. However, we did not observe any severe impairment in mitochondrial function in the pre-manifest HDEMCs. It remains to be confirmed at which stage of the disease the mitochondrial involvement can be reliably detected at the level of whole mitochondria. However, the major advantage of our technique is that it allows to detect a disturbance of mitochondrial respiration close to the in-vivo condition using our homogenized samples. Therefore, we hypothesize that alterations of mitochondrial function may be reliably detected at later stages of the disease.

For processing the muscle samples for respiratory measurements homogenization of the tissue was performed. Compared to the measurement of isolated mitochondria in such a homogenate all mitochondria are measured. During the isolation procedure often distinct populations of mitochondria are selected while fragile or potentially damaged mitochondria can get lost. An even more advantageous way to measure muscle tissue by HRR would be to permeabilize isolated muscle fibers, since permeabilization was described to be beneficial compared to homogenization for HRR [38]. For our experiments we preferred to homogenize the human muscle tissue because the very small tissue biopsies obtained by our technique are more difficult to divide into single fibers for permeabilization. In addition some fibers were damaged during the process of the biopsy impeding to guarantee for intact fibers and to control for comparable conditions between the measurements. Therefore, we decided to homogenize muscle samples to guarantee homogeneity and comparability between different samples and yielding amongst others a higher cytochrome c response when testing for membrane integrity [38]. Increasing respiration upon cytochrome c administration was detected with a mean increase of 23.9% (SD±9.8) for the human muscle samples. Such an increase in the cytochrome c response has already been described in a comparative analysis of different permeabilization and homogenization methods for HRR [38]. In permeabilized muscle fibers, the response to cytochrome c seems to be lower, but this technique cannot be applied to other tissues like liver or brain, which we studied in mice.

The knock-in HD mouse model Hdh^Q111^ was chosen to put our results in context with our human data. The expression of the full-length huntingtin under control of the human promoter may serve as an adequate model for pre-manifest HDEMCs. The mice used in our study were on average 471 days old, representing 15 months of age. At this age the Hdh^Q111^ were phenotypically indistinguishable from corresponding controls and wt mice, although nuclear inclusions of huntingtin are evident in the striatal neurons at 12 months of age, as described previously [39]. Therefore, the mice used in our study represent the clinical stage of human pre-manifest HDEMCs. In the Hdh^Q111^ mice the nuclear inclusions in the striatum were reported to induce motor dysfunction and gait deficits at the age of 24 months. For further studies examining human early manifest HDEMCs it would be beneficial to include 24 months old Hdh^Q111^ mice as corresponding mouse model. Indeed, in agreement to our findings in humans the mitochondrial respiratory activity in the soleus muscle of the Hdh^Q111^ mice did not show differences. In contrast, liver and cortex of Hdh^Q111^ mice showed tissue specific differences in the mitochondrial activity, thus demonstrating the sensitivity of the methods employed to disclose respiratory chain activity alterations once present. Normalization of the respiratory data to the CS activity showed no significant differences between Hdh^Q111^ and Hdh^Q20^ mice. When the results were shown as ratio of the respiration to the *mU* capacity the cortex of Hdh^Q111^ mice showed significantly decreased respiratory levels for *C*_*II*_ and *mO* compared to Hdh^Q20^ mice. The same results were reported in the striatum of R6/2 mice before [40].

The CS activity is one measure for the mitochondrial content and normalization of the respiratory data to this value allowed to examine the respiration of the tissue independent of the mitochondrial content. The mitochondrial mass can be influenced by factors like physical activity or aging and it was shown that reduced mitochondrial content is related to age-related muscle mitochondrial dysfunction [41]. As second marker for the mitochondrial mass the mtDNA copy number was determined. Our experiments showed the same tendency of both markers, but they were not equivalent. Previous studies already described the CS activity as a more precise marker for the mitochondrial mass compared to the mtDNA copy number, because the latter mentioned can vary considerably between individuals [42].

Most previous studies in HD mouse models showing a compromised mitochondrial function focused on the CNS [27, 40, 43]. Few reports showed in addition mitochondrial alterations in peripheral organs like liver [44] or heart [45]. In the case of the liver, more recent human data presented by Stüwe et al., which measured ^13^C exhalation as a marker of methionine metabolism in HD patients, are consistent with a mitochondrial dysfunction in liver of HD patients [5]. All these reports suggest an ubiquitous decrease in mitochondrial function in presence of mHTT, but the experimental approaches applied for the respective investigations differed substantially, thus rendering a conclusive comparison of data from different groups challenging. Therefore, for our study in parallel mitochondrial respiration in frontal cortex, soleus muscle, liver and heart of one individual Hdh^Q111^ mouse was measured in parallel by means of high-resolution respirometry, thus maximizing the comparability of data from distinct tissues.

We found a decreased respiratory activity in the liver under maximum OxPhos and selective complex II stimulation. This result is particularly important in the context of the specific metabolic symptoms of HD, albeit these actual data obviously cannot show how the reduced mitochondrial activity observed in the liver could be precisely linked to specific symptoms of HD, for example to the characteristic weight loss. Most importantly, our results support the findings by Stüwe et al. [5, 46]. In fact, methionine metabolism in the liver is directly linked to the activity of the mitochondrial respiratory chain, which could be therefore the limiting factor in this case. In contrast to the liver, we did not find an altered mitochondrial activity in the heart. Previous data suggesting that the heart is probably involved in the pathology of HD were mainly obtained in mouse models. For example, Mihm et al. observed a cardiac dysfunction in the R6/2 mouse [45, 47]. In the Hdh^Q111^ mouse some minor alterations in the cardiovascular function were also observed, but the available data do not allow to discriminate between possible causes [48]. A potential link to mitochondrial function in cardiac tissue was not further investigated in this mouse model so far. Our present data exclude major changes in mitochondrial respiration in the myocardium, suggesting that other causes may be more likely to affect the cardio-circulatory physiology of Hdh^Q111^ mice.

In conjunction with our human data, the results in the skeletal muscle and prefrontal cortex of the Hdh^Q111^ mouse model are of particular importance. In agreement with our findings in the HDEMCs, in the soleus muscle we did not observe any difference in mitochondrial respiration between disease model and control. In this context we would like to point out that different muscles with diverse muscle fiber type composition were examined in our study. For the human fine-needle muscle biopsies and the murine qPCR data the quadriceps was examined, which consists of a mixed oxidative and glycolytic fibers. The muscle soleus was chosen for the murine studies due to its homogeneous composition of a fast-twitch fiber type, which are also called oxidative fibers. The murine muscle soleus is easily isolated and it includes a high mitochondrial content. A direct comparison of the respiratory results gained from the human vastus lateralis muscle and the murine soleus muscle is therefore not possible. It has previously been shown that mitochondrial respiration differs between murine soleus muscle compared to the murine quadriceps and gastrocnemius muscle [49]. Furthermore, the murine quadriceps muscle has been shown to be a suitable model for the human skeletal muscle [49]. Due to the high mitochondrial mass of the soleus muscle the respiratory values depend on the mitochondrial content, which should be considered for analysis. Surprisingly, mitochondrial activity in the prefrontal cortex was even increased in the Hdh^Q111^ mouse compared to the Hdh^Q20^ controls, albeit the difference was rather small. This result clearly contradicts that changes of mitochondrial function as found in the skeletal muscle generally mirror those in the CNS.

Noteworthy, another study in the R6/2 mouse model showed an increased mitochondrial complex activity in whole brain and striatum, which is in accordance with our findings, but a decreased one in cortex. Normalized to CS activity the respiratory activity was increased in the transgenic mice [27]. These data as well as our current results seem to contradict a study by Aidt et al., where the striata of the R2/6 HD mouse model showed decreased maximum mitochondrial activity using high-resolution respirometry [40]. The selective enzymatic activities of respiratory chain complexes as measured by Hering et al. do not necessarily reflect the function of the respiratory chain in the physiological environment of whole mitochondria. Cortex was chosen in our study because it is affected in HD [50, 51] and in contrast to the striatum it is not the primary target of excitotoxic mechanisms [52, 53]. Compared to our study Aidt et al. examined the striatum, which is a more vulnerable region concerning neuronal loss and dysfunction in HD compared to the cortex, partially explaining the contrasting results [40].

In all investigated tissues of our present study, the mRNA levels of key enzymes involved in mitochondrial biogenesis like *Nrf1*, *Ppargc1a* and *Tfam* showed no changes between Hdh^Q111^ mice and Hdh^Q20^ controls. In accordance with this finding, the CS activity and mtDNA copy number determined in the tissue samples in parallel to the high-resolution respirometry were unaltered between the two groups. Therefore, changes of the mitochondrial mass as a theoretically possible explanation for the alterations in mitochondrial respiration observed in liver and cortex can be excluded. Interestingly, we observed reduced mRNA levels of the ROS detoxifying enzyme SOD2 in the cortex of Hdh^Q111^ mice. Since we did not measure ROS production, a functional interpretation of this result in terms of a modified level of oxidative stress is not possible. Nevertheless, according to the “uncoupling to survive” hypothesis [54] the simultaneously increased mRNA levels of the uncoupling protein *Ucp2* could represent a compensatory mechanism. In fact, an increased exposure to superoxide radicals in the mitochondria as consequence of the reduced SOD2 levels, could be mitigated by uncoupling through activation of the proton conductance of UCPs [55].

In conclusion, our data provide evidence that the respiratory function of intact skeletal muscle mitochondria is unchanged in pre-manifest HDEMCs compared to controls. This finding does not necessarily contradict previous results showing that some aspects of mitochondrial function may be measurably altered at this stage of HD, but they suggest that these alterations are not yet compromising the normal respiratory activity of whole mitochondria at rest. Furthermore, we show that our technique is well tolerated and allows to obtain valuable materials for detailed studies on mitochondrial respiration or other biochemical parameters in human skeletal muscle tissue without imposing unacceptable burdens to patients. Since mitochondrial defects are expected to become manifest at later stages of the disease, additional experiments with symptomatic HDEMCs may identify stage-dependent changes of mitochondrial function in patients.

Our data cautions against the notion that skeletal muscle tissue necessarily reflects the functional properties of tissues from other organs with regard to mitochondrial respiration. Therefore, we cannot exclude that alterations in mitochondrial respiratory activity could be already detectable in the CNS or other peripheral tissues (e.g. liver) from pre-manifest HDEMCs, although they are not measurable in the skeletal muscle.

## Supporting information

S1 Fig HRR of human muscle biopsies normalized to CS, mU and COX. High-resolution respirometry analysis of human vastus lateralis muscle biopsies. The white boxes represent human controls versus HD expansion mutation carriers (HDEMC) (gray boxes). (A) The respiratory values for complex I activity (CI), complex II activity (CII), the maximum OxPhos capacity (mO), the maximum uncoupled capacity (mU) and the O2ATP (calculated oxygen consumption linked to ATP production) are shown normalized to the respective citrate synthase (CS) activity. Leak respiration (B) and the respiratory control ratio (RCR) (C) are given normalized to the CS activity for the respective samples, where the CS activity was available. For CS activity data n = 7 controls and n = 4 HDEMCs. n = 9 controls, n = 7 HDEMCS were used. The respiratory data normalized to the *mU* (D) and the cytochrome c oxidase (COX) activity (E) were calculated. Controls n = 9, HDEMCs n = 7, Mann-Whitney test comparing controls vs. HDEMCs, ns = P>0.05.
(TIF)

S2 Fig HRR of murine cortex, liver, m. soleus and heart normalized to CS activity and mU. High resolution respirometry in prefrontal cortex, liver, soleus muscle and heart of Hdh^Q20^ and Hdh^Q111^ mice. The white boxes represent Hdh^Q20^ control mice versus the gray boxes, which represent the HD mouse model Hdh^Q111^. The respiratory values determined are normalized to the respective citrate synthase (CS) activity of each sample (A-E). (A) Complex I activity (C_I_) is shown in a coupled state determined after addition of ADP. (B) Complex II activity (C_II_) was measured in an uncoupled state after addition of rotenone. (C) The *maximum OxPhos* capacity was measured and (D) the *maximum uncoupled capacity* was determined after application of FCCP in all four tissues. (E) For murine cortex and liver the oxygen consumption linked to ATP production (O_2_ATP) was calculated. All respiratory values determined were normalized to the mU (F-I) to determine the flux control ratio. n = 6, Mann-Whitney test comparing genotypes, ns = p>0.05, * = p≤0.05, ** = p≤0.01.
(TIF)

S1 Table Human data. Raw values of the High resolution respirometry experiments and the citrate synthase activity are provided.
(XLSX)

S2 Table HdhQ mice HRR. Raw values of the High resolution respirometry experiments are provided including normalization of the data.
(XLSX)

S3 Table HdhQ mice mt DNA copy number. Raw values of the qPCR experiments to determine the mitochondrial DNA copy number.
(XLSX)

S4 Table HdhQ mice citrate synthase analysis. Raw values of the citrate synthase activity assay are provided.
(XLSX)

S5 Table HdhQ mice mRNA analysis. Raw values of the qPCR experiments are provided including expression analysis of mitochondrial targets.
(XLSX)
