# Supplementary figures and images for: High-resolution respirometry of fine-needle muscle biopsies in pre-manifest Huntington’s disease expansion mutation carriers shows normal mitochondrial respiratory function
Source: PLoS One. 2017 Apr 13;12(4):e0175248. doi: 10.1371/journal.pone.0175248 (PMC5390997; doi:10.1371/journal.pone.0175248)

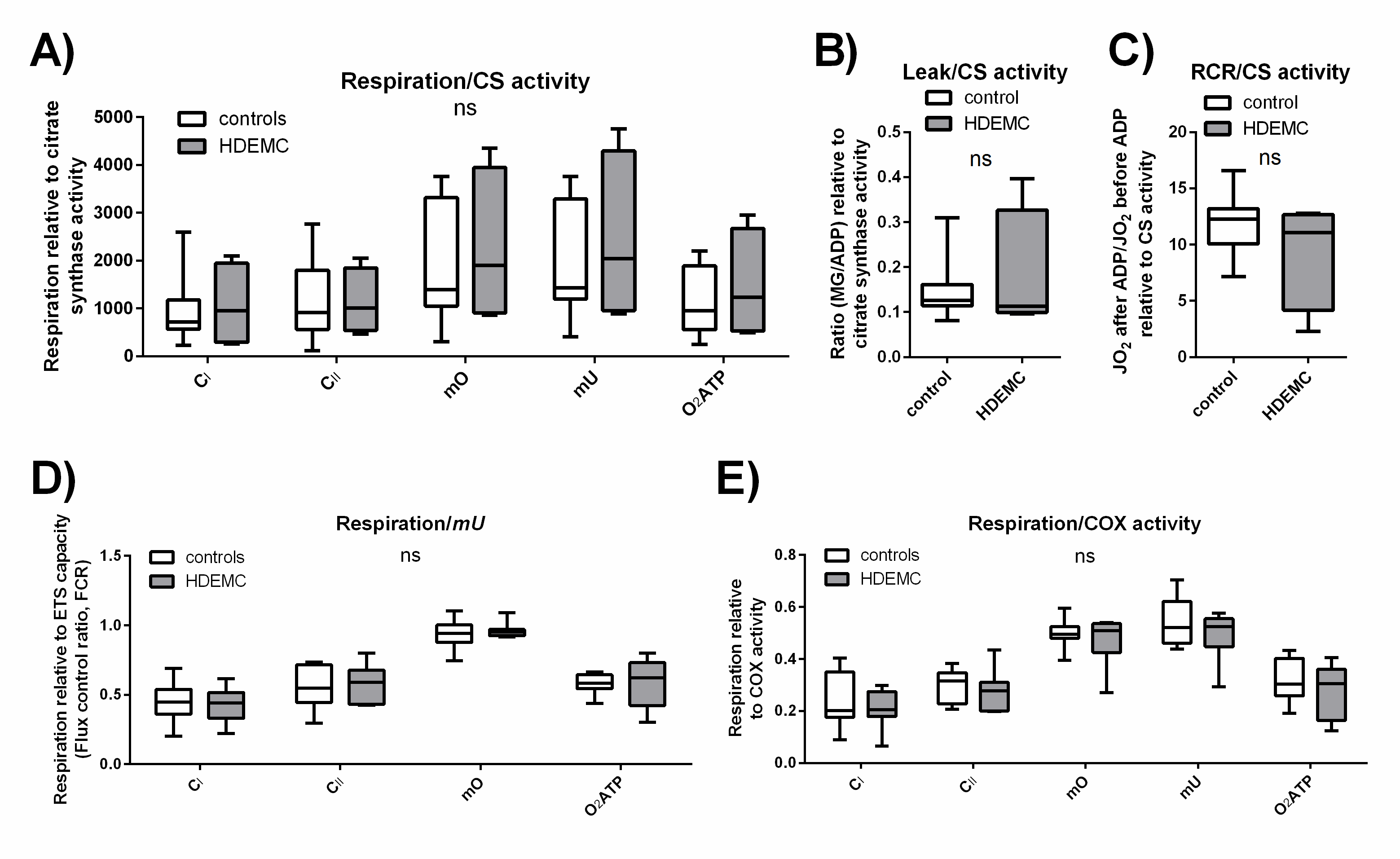

Supplement: S1 Fig — High-resolution respirometry analysis of human vastus lateralis muscle biopsies. The white boxes represent human controls versus HD expansion mutation carriers (HDEMC) (gray boxes). (A) The respiratory values for complex I activity (CI), complex II activity (CII), the maximum OxPhos capacity (mO), the maximum uncoupled capacity (mU) and the O2ATP (calculated oxygen consumption linked to ATP production) are shown normalized to the respective citrate synthase (CS) activity. Leak respiration (B) and the respiratory control ratio (RCR) (C) are given normalized to the CS activity for the respective samples, where the CS activity was available. For CS activity data n = 7 controls and n = 4 HDEMCs. n = 9 controls, n = 7 HDEMCS were used. The respiratory data normalized to the mU (D) and the cytochrome c oxidase (COX) activity (E) were calculated. Controls n = 9, HDEMCs n = 7, Mann-Whitney test comparing controls vs. HDEMCs, ns = P>0.05. (TIF) [file pone.0175248.s001.tif]

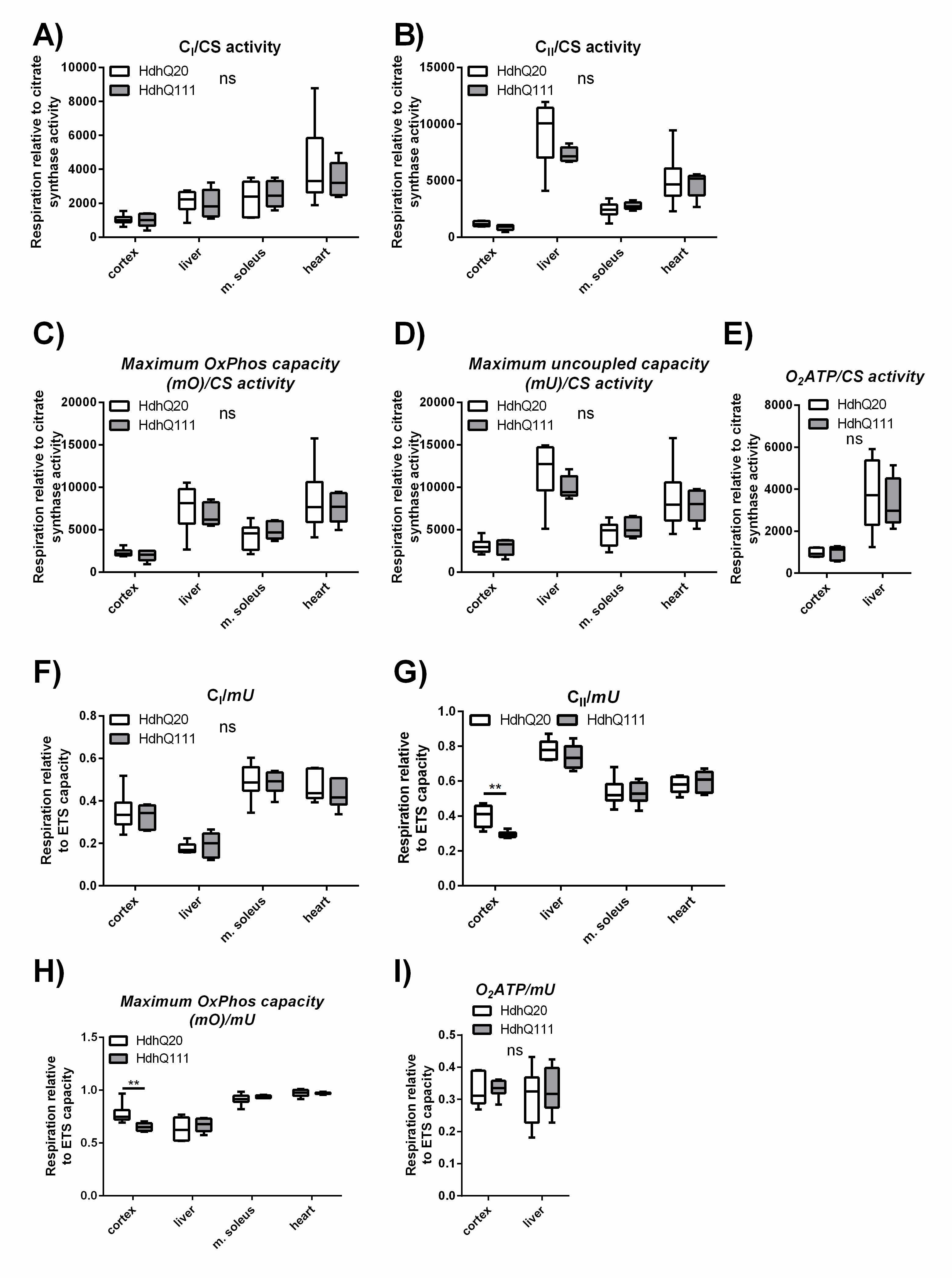

Supplement: S2 Fig — High resolution respirometry in prefrontal cortex, liver, soleus muscle and heart of HdhQ20 and HdhQ111 mice. The white boxes represent HdhQ20 control mice versus the gray boxes, which represent the HD mouse model HdhQ111. The respiratory values determined are normalized to the respective citrate synthase (CS) activity of each sample (A-E). (A) Complex I activity (CI) is shown in a coupled state determined after addition of ADP. (B) Complex II activity (CII) was measured in an uncoupled state after addition of rotenone. (C) The maximum OxPhos capacity was measured and (D) the maximum uncoupled capacity was determined after application of FCCP in all four tissues. (E) For murine cortex and liver the oxygen consumption linked to ATP production (O2ATP) was calculated. All respiratory values determined were normalized to the mU (F-I) to determine the flux control ratio. n = 6, Mann-Whitney test comparing genotypes, ns = p>0.05, * = p≤0.05, ** = p≤0.01. (TIF) [file pone.0175248.s002.tif]
